# Influence of Impacted Mandibular Third Molar Position on the Clinical and Radiographic Periodontal Status of Adjacent Second Molars: A Cross-Sectional Study

**DOI:** 10.7759/cureus.113095

**Published:** 2026-07-21

**Authors:** Halil Ibrahim Durmus, Kagan Fatih Kusan, Tevfik Kizilseki, Hasan Demirkol, Kaan Mert Sekerci, Adnan Oztas

**Affiliations:** 1 Oral and Maxillofacial Surgery, Faculty of Dentistry, Adiyaman University, Adiyaman, TUR; 2 Oral and Maxillofacial Surgery, Adiyaman University, Adiyaman, TUR

**Keywords:** alveolar bone loss, impacted third molar, pell and gregory classification, periodontal pocket depth, second molar, winter classification

## Abstract

Aim

The aim of this study is to evaluate the effect of positional characteristics of mandibular impacted third molars on the clinical and radiographic periodontal status of adjacent second molars.

Materials and methods

This single-center, analytical cross-sectional study was conducted at the Department of Oral and Maxillofacial Surgery, Faculty of Dentistry, Adiyaman University. Fifty patients (100 third molar sites) with bilateral mandibular impacted third molars were included. Systemically healthy individuals aged 15-40 years without periodontitis were evaluated. Periodontal pocket depths at the distal aspects of the mandibular second molars were measured using a standard periodontal probe, and distal alveolar bone loss was assessed on panoramic radiographs. Winter angulation, Pell and Gregory classification, and impaction status were recorded. Statistical analyses included the Shapiro-Wilk test, Mann-Whitney U test, Kruskal-Wallis test, Spearman correlation analysis, multivariable linear regression, and receiver operating characteristic (ROC) curve analysis. Statistical significance was set at p < 0.05.

Results

Univariate analyses showed no significant associations between third molar position characteristics and periodontal parameters (p>0.05). Multivariate linear regression identified only Pell-Gregory Class III impaction and distal alveolar bone loss as independent predictors of maximum periodontal pocket depth (p<0.05). Maximum periodontal pocket depth was positively correlated with distal alveolar bone loss. ROC analysis demonstrated acceptable discrimination for predicting periodontal pocket depth ≥4 mm (area under the curve (AUC) = 0.709).

Conclusion

Impacted mandibular third molar position alone does not significantly affect the periodontal status of adjacent second molars. Distal alveolar bone loss is an independent predictor of periodontal pocket depth and may support treatment planning.

## Introduction

Mandibular third molars are the teeth most likely to remain impacted in the mouth due to being the last teeth to complete tooth development and having insufficient eruption space [1]. Impacted third molars are associated with various pathologies such as pericoronitis, odontogenic infections, caries formation, odontogenic cysts, root resorption in adjacent teeth, and periodontal diseases [2-5]. However, whether or not to extract asymptomatic impacted third molars prophylactically is still a controversial issue, and the long-term periodontal health of the adjacent second molar is considered an important determinant in this discussion [2,6]. It is reported that mandibular impacted third molars increase plaque retention on the distal surface of the second molar, making it difficult to maintain oral hygiene, and consequently, can cause gingival inflammation, periodontal pocket formation, and alveolar bone loss [7-8]. Periodontal destruction developing in the distal region can negatively affect the prognosis of the second molar over time and can lead to periodontal complications that may progress to tooth loss in advanced cases [9,4]. Therefore, the evaluation of the periodontal effects caused by third molars is considered one of the important clinical criteria in determining the extraction indication [2,6,10].

It has been shown that the periodontal effect of an impacted third molar on the adjacent second molar may vary depending on the characteristics of the impaction. In particular, it has been reported that mesioangular and horizontally impacted third molars, according to the Winter classification, cause greater periodontal destruction by increasing plaque retention and food accumulation [11,12]. Similarly, it has been noted that, according to the Pell and Gregory classification, impacted third molars located at a deeper level or in a more posterior position within the ramus may increase distal periodontal pocket depth and alveolar bone loss [12,13]. However, the current literature reports conflicting findings regarding the strength of this association and which positions carry a higher risk.

Although numerous studies in the literature evaluate the periodontal effects of impacted third molars on adjacent second molars, a significant portion of these studies have only used clinical periodontal measurements or only radiographic evaluations [8,11,13]. Studies that evaluate clinical periodontal probing findings together with the distal alveolar bone level obtained from panoramic radiographs are limited. The combined evaluation of clinical and radiographic parameters may contribute to a more comprehensive understanding of the periodontal effects of impacted third molars. Therefore, the aim of this study is to evaluate the relationship between the positional characteristics of impacted third molars and the periodontal pocket depth and radiographic distal alveolar bone level of adjacent second molars in individuals with bilateral mandibular impacted third molars who applied to the Department of Oral, Dental and Maxillofacial Surgery, Adiyaman University Faculty of Dentistry.

## Materials and methods

Study design and patient selection

This study was planned as a single-center, observational, analytical cross-sectional study conducted at the Department of Oral and Maxillofacial Surgery, Faculty of Dentistry, Adiyaman University. This study was reported in accordance with the Strengthening the Reporting of Observational Studies in Epidemiology (STROBE) guidelines [14]. Ethical approval was obtained from the Adiyaman University Non-Interventional Clinical Research Ethics Committee (Decision No: 2026/4-3, Date: 20.05.2026). Fifty patients with bilateral mandibular impacted third molars were included in the study. To minimize the confounding effect of age-related physiological periodontal changes and generalized alveolar bone loss, only individuals aged 15-40 years who were systemically healthy, had no diagnosis of periodontitis on periodontal examination, and had no history of periodontal treatment were included. Patients with restorations, crowns, root canals, caries, or advanced periodontal destruction in the mandibular second molar, as well as those with caries in the adjacent third molar, were excluded from the study.

Demographic data (age and sex), along with third molar angulation according to the Winter classification and the Pell and Gregory classification, as previously described in the literature [1,9], and impaction status, were recorded. The distal periodontal status of the right and left mandibular second molars was clinically assessed in each patient. Periodontal pocket depth measurements were performed using a Kohler CP15 periodontal probe (Item 3197; Kohler Medizintechnik GmbH, Stockach, Germany), a diagnostic periodontal instrument with continuous 1-15 mm millimeter markings that allows accurate periodontal pocket depth measurements and is suitable for comprehensive periodontal examination. Clinical periodontal examination was performed according to the diagnostic principles of the 2017 World Workshop on the Classification of Periodontal and Peri-Implant Diseases and Conditions [15]. Measurements were obtained by applying gentle probing force with the probe parallel to the long axis of the tooth until the base of the periodontal pocket, and values were recorded to the nearest millimeter. Periodontal pocket depths were measured separately at the distobuccal, distal, and distolingual aspects of each mandibular second molar. To minimize observer-induced variability, all clinical assessments were performed by the same investigator using the same periodontal probe. Owing to the study design, periodontal probing measurements were not blinded to the radiographic findings.

The positional characteristics of the mandibular third molars were evaluated using panoramic radiographs. Third molar angulation was classified according to the Winter classification, whereas the relationship to the mandibular ramus and the occlusal plane was classified according to the Pell and Gregory classification, as previously described in the literature [1,9,16]. In addition, third molars were categorized as completely or partially impacted according to their eruption status.

Radiographic imaging and image analysis

**Figure 1:**
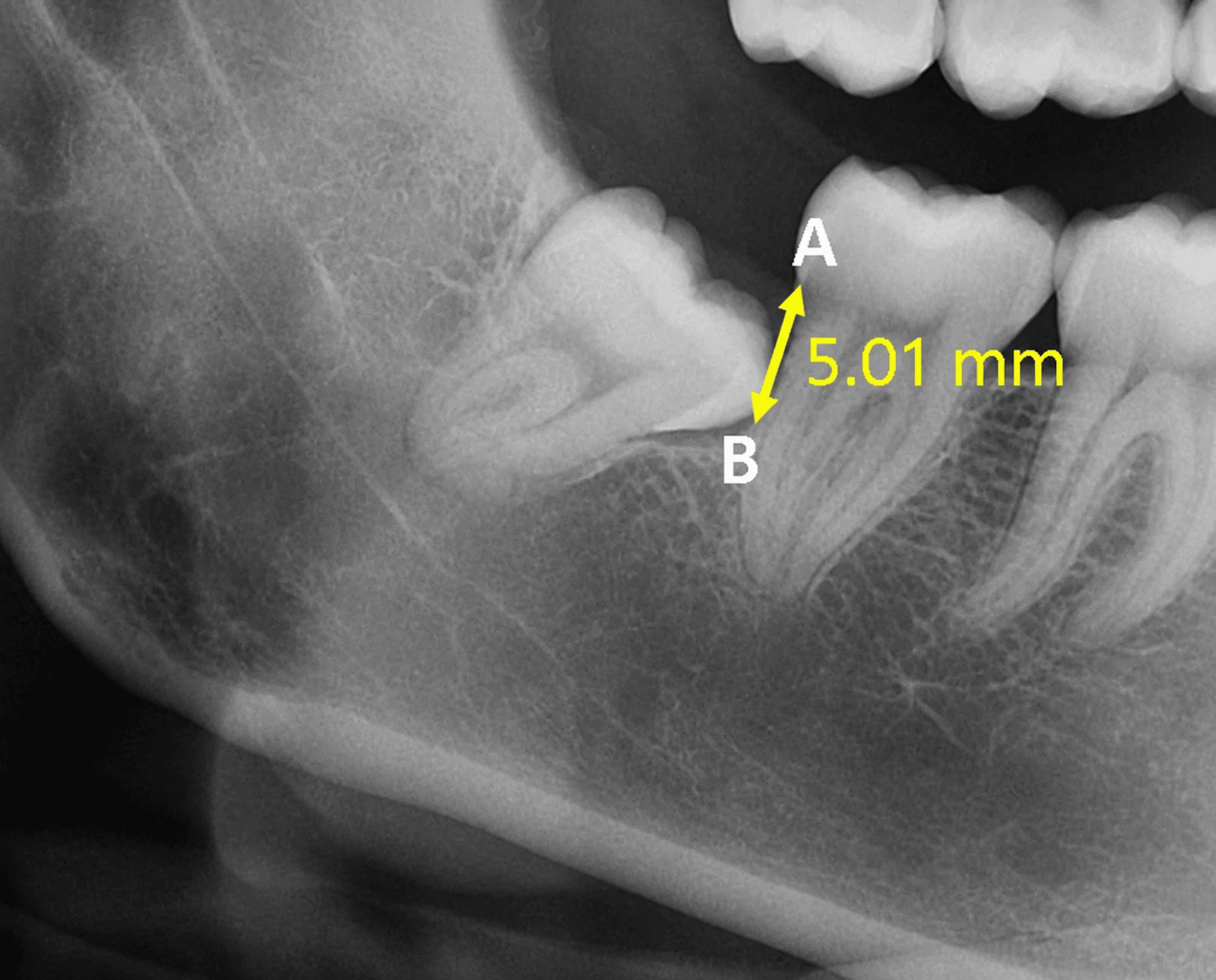
Measurement of the distal alveolar bone level on a panoramic radiograph. A: Distal cementoenamel junction (CEJ) of the mandibular second molar. B: Most coronal point of the distal alveolar crest. The measurement was performed as the linear distance between points A and B.

All panoramic radiographs were obtained by an experienced radiology technician using the same digital panoramic imaging system with standard imaging parameters (65 kVp, 5 mA, 17 seconds). Patient positioning was performed according to the manufacturer's recommendations, and care was taken to ensure standardization in all images. Radiographs were exported in uncompressed TIFF format at 300 dpi resolution. Radiographic evaluations were performed on the digital panoramic images. The distal alveolar bone level of the mandibular second molar was determined by measuring the linear distance in millimeters between the distal enamel-cement junction (CEJ) of the second molar and the most coronal point of the distal alveolar crest (Figure 1). Measurements were performed using the calibrated linear measurement tool of the panoramic imaging software, and the obtained values ​​were rounded to the nearest 0.5 mm for standardization purposes. All radiographic measurements were performed by the same researcher. To minimize measurement bias, all clinical and radiographic assessments were performed by the same investigator using standardized examination protocols.

In the study, age, sex, side, angulation of the third molar, Pell and Gregory classifications, impaction status, and distobuccal, distal, and distolingual periodontal pocket depths, as well as the radiographic distal alveolar bone level, were recorded as study variables.

Statistical analysis

Statistical analyses were performed using IBM SPSS Statistics for Windows, Version 26.0 (IBM Corp., Armonk, NY, USA). The normality of continuous variables was assessed using the Shapiro-Wilk test. For non-normal distributions, the Mann-Whitney U test was used to compare two groups, and the Kruskal-Wallis test was used to compare three or more groups. Appropriate post hoc comparisons were performed in cases where significant differences were found. The relationship between continuous variables was evaluated using Spearman rank correlation analysis. The performance of distal alveolar bone loss in predicting a periodontal pocket depth of ≥4 mm was examined using receiver operating characteristic (ROC) curve analysis, and diagnostic accuracy was evaluated using the area under the curve (AUC). Multivariate linear regression analysis was performed to identify independent factors affecting maximum periodontal pocket depth. The regression model included age, gender, Winter angulation, Pell and Gregory classification, impaction status, and distal alveolar bone loss variables.

Since both the right and left mandibular third molar regions were evaluated in each participant, the potential dependence of bilateral observations was considered when interpreting the statistical findings. The statistical power of the study was evaluated using post hoc power analysis based on the observed effect size. In the post hoc power analysis, the statistical power of the study was calculated as 94.6% at a 5% significance level (α = 0.05). In all statistical analyses, a p < 0.05 value was considered statistically significant. 

## Results

The study included 50 patients with bilateral mandibular impacted third molars, and a total of 100 mandibular third molar regions were evaluated. The mean age of the patients was 24.7 ± 6.3 years (range: 15-40 years). Most of the evaluated third molars were classified as having vertical angulation (64.0%), Pell-Gregory Class I (45.0%), and Position A (54.0%). Additionally, 60.0% of the third molars were partially impacted, while 40.0% were fully impacted (Table 1).

**Table 1:** Demographic and clinical characteristics of the study sample

Variable	Value
Number of patients	50
Number of evaluated mandibular third molars	100
Age (years), mean ± SD	24.7 ± 6.3
Age range (years)	15-40
Male (third molars)	53 (53.0%)
Female (third molars)	47 (47.0%)
Right side	50 (50.0%)
Left side	50 (50.0%)
Winter angulation	
Vertical	64 (64.0%)
Mesioangular	24 (24.0%)
Horizontal	9 (9.0%)
Distoangular	3 (3.0%)
Pell-Gregory class	
Class I	45 (45.0%)
Class II	35 (35.0%)
Class III	20 (20.0%)
Pell-Gregory position	
Position A	54 (54.0%)
Position B	36 (36.0%)
Position C	10 (10.0%)
Impaction status	
Complete impaction	40 (40.0%)
Partial impaction	60 (60.0%)

The model created in the multivariate linear regression analysis was found to be statistically significant (R² = 0.212, adjusted R² = 0.113, F = 2.148, p = 0.025). Distal alveolar bone loss was found to be an independent predictor of maximum periodontal pocket depth (β = 0.464, 95% CI: 0.149-0.780, p = 0.004). Furthermore, Pell-Gregory Class III impaction was found to be significantly associated with maximum periodontal pocket depth (β = −1.367, 95% CI: −2.634 - −0.100, p = 0.035). No significant association was found between age, gender, Winter angulation, Pell-Gregory position, and impaction status and maximum periodontal pocket depth (p > 0.05) (Table 5). The mean periodontal pocket depths measured in the mandibular second molars were found to be 2.77 ± 1.37 mm in the distobuccal region, 2.41 ± 1.47 mm in the distal region, and 2.59 ± 1.30 mm in the distolingual region, respectively. The mean maximum periodontal pocket depth was 3.25 ± 1.60 mm, and the mean distal alveolar bone loss measured in panoramic radiographs was 2.88 ± 1.02 mm (Table 2). 

**Table 2:** Clinical periodontal and radiographic measurements of the adjacent mandibular second molars

Variable	Mean ± SD	Median	Minimum	Maximum
Distobuccal probing depth (mm)	2.77 ± 1.37	2.0	1	7
Distal probing depth (mm)	2.41 ± 1.47	2.0	0	7
Distolingual probing depth (mm)	2.59 ± 1.30	2.0	1	7
Maximum probing depth (mm)	3.25 ± 1.60	3.0	1	7
Distal alveolar bone loss (mm)	2.88 ± 1.02	3.0	1	5

No statistically significant association was found between Winter angulation of mandibular third molars, Pell-Gregory class, Pell-Gregory position, and impaction status and maximum periodontal pocket depth or distal alveolar bone loss (all p > 0.05) (Table 3). However, the association between Winter angulation and distal alveolar bone loss was found to be near statistical significance (p = 0.082). 

**Table 3:** Comparison of maximum periodontal probing depth and distal alveolar bone loss according to mandibular third molar position characteristics P-values were calculated using the Mann-Whitney U test or Kruskal-Wallis test, as appropriate.

Comparison	Maximum probing depth (p)	Distal alveolar bone loss (p)
Winter angulation	0.176	0.082
Pell-Gregory class	0.249	0.845
Pell-Gregory position	0.942	0.981
Complete vs. partial impaction	0.798	0.322

**Figure 2:**
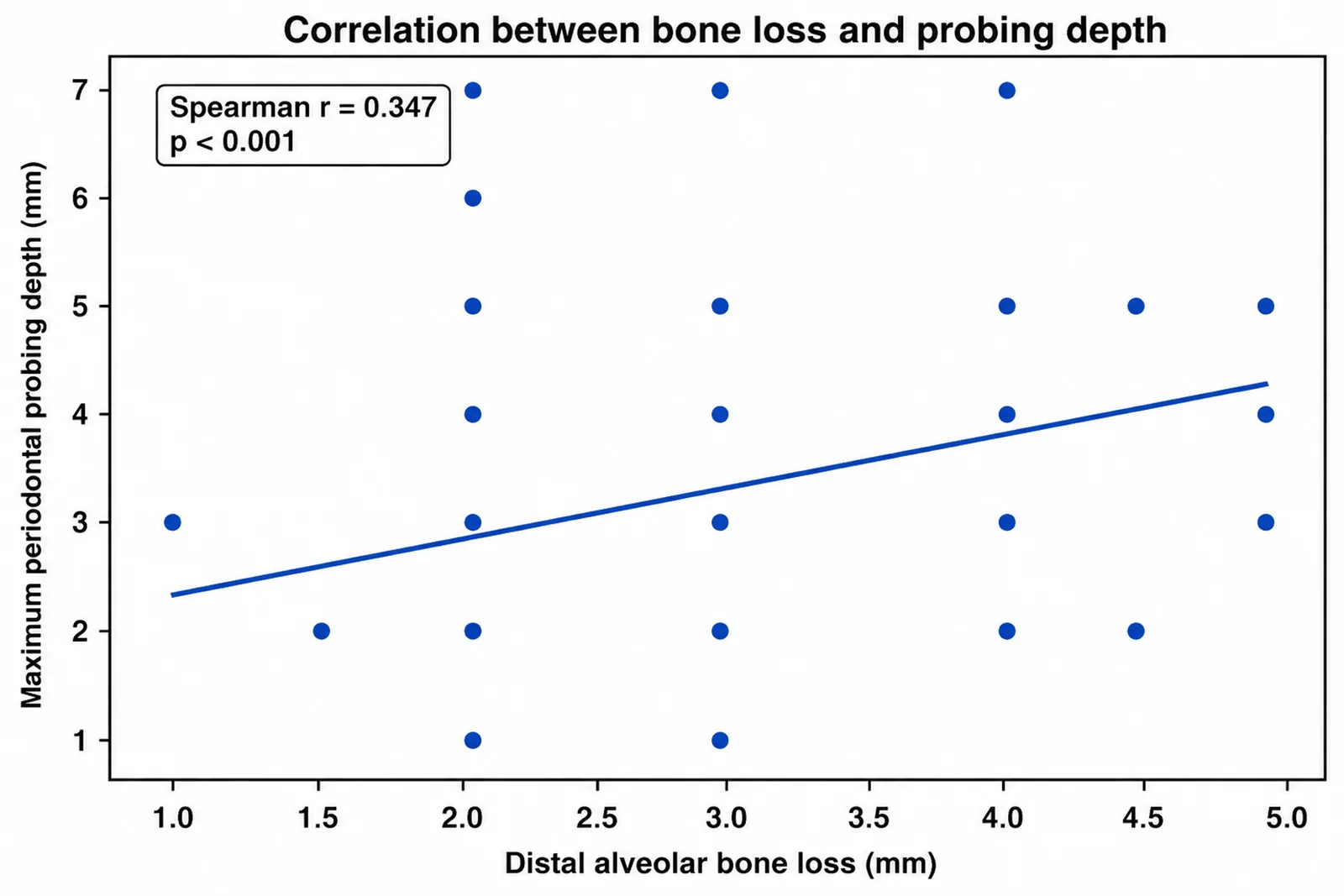
Correlation between distal alveolar bone loss and maximum periodontal probing depth in mandibular second molars adjacent to impacted third molars.

Spearman correlation analysis revealed a moderately positive and statistically significant association between maximum periodontal pocket depth and distal alveolar bone loss (r = 0.347, p < 0.001). As distal alveolar bone loss increased, the maximum periodontal pocket depth was also observed to increase (Table 4, Figure 2).

**Table 4:** Correlation analysis between maximum probing depth and distal alveolar bone loss

Variable	Spearman's r	p
Maximum probing depth × Distal alveolar bone loss	0.347	<0.001

The model created in the multivariate linear regression analysis was found to be statistically significant (R² = 0.212, adjusted R² = 0.113, F = 2.148, p = 0.025). Distal alveolar bone loss was found to be an independent predictor of maximum periodontal pocket depth (β = 0.464, 95% CI: 0.149-0.780, p = 0.004). Furthermore, Pell-Gregory Class III impaction was found to be significantly associated with maximum periodontal pocket depth (β = −1.367, 95% CI: −2.634 - −0.100, p = 0.035). No significant association was found between age, gender, Winter angulation, Pell-Gregory position, and impaction status and maximum periodontal pocket depth (p > 0.05) (Table 5). 

**Table 5:** Multivariable linear regression analysis for maximum periodontal probing depth Model statistics: R² = 0.212, Adjusted R² = 0.113, F = 2.148, Model p = 0.025

Variable	β	95% CI	p
Age	0.038	−0.015 to 0.091	0.160
Female sex	0.240	−0.384 to 0.863	0.447
Horizontal angulation	1.762	−0.389 to 3.912	0.107
Mesioangular angulation	0.811	−1.094 to 2.716	0.400
Vertical angulation	1.410	−0.392 to 3.213	0.124
Pell-Gregory Class II	−0.070	−0.807 to 0.666	0.850
Pell-Gregory Class III	−1.367	−2.634 to −0.100	0.035
Pell-Gregory Position B	−0.488	−1.262 to 0.287	0.214
Pell-Gregory Position C	0.375	−1.148 to 1.897	0.626
Partial impaction	−0.594	−1.448 to 0.260	0.170
Distal alveolar bone loss	0.464	0.149 to 0.780	0.004

**Figure 3:**
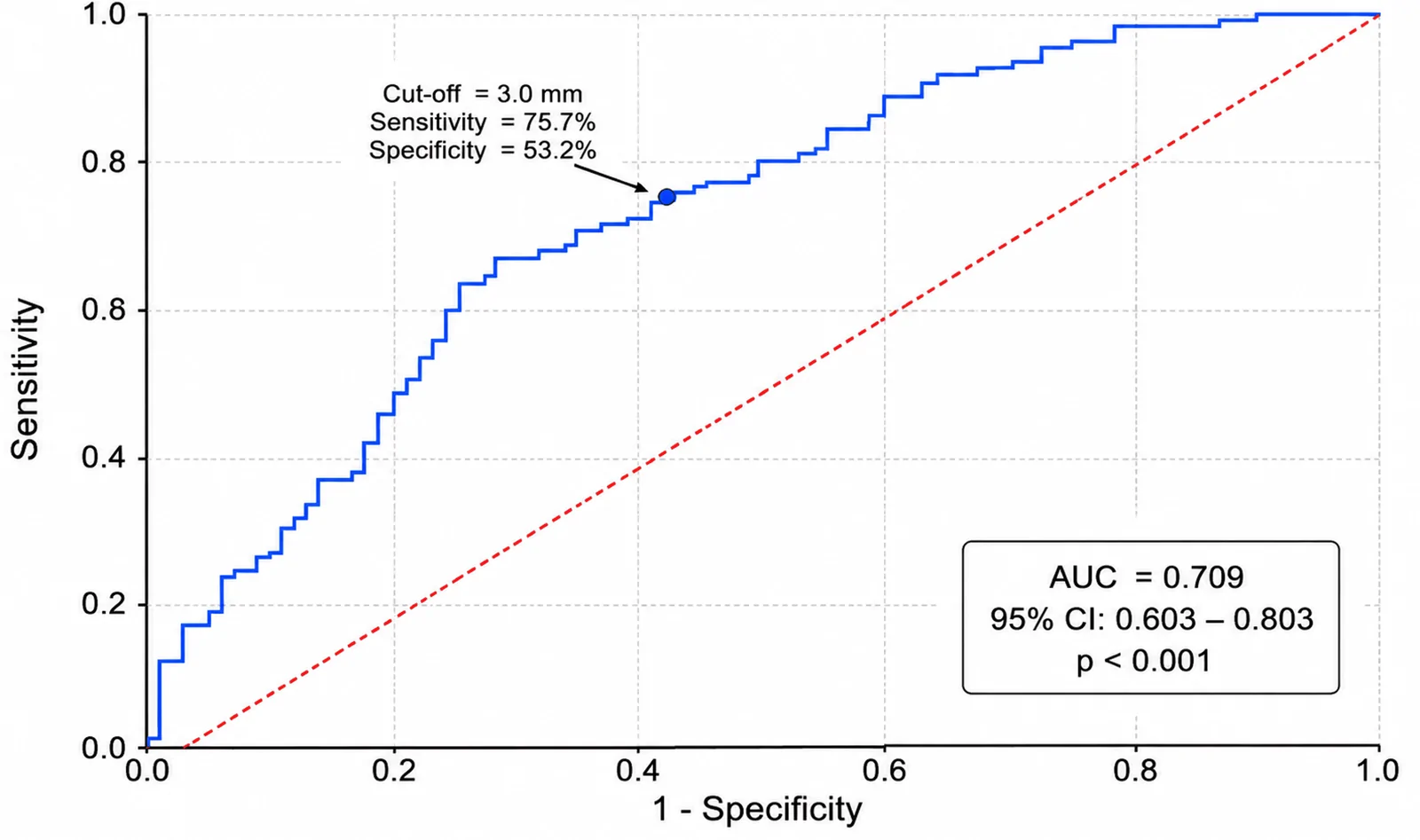
Receiver operating characteristic (ROC) curve showing the diagnostic performance of distal alveolar bone loss for predicting a maximum periodontal probing depth of ≥4 mm (AUC = 0.709). AUC: area under the curve

In the ROC analysis, distal alveolar bone loss was found to demonstrate acceptable discriminatory performance in predicting a maximum periodontal pocket depth of ≥4 mm (AUC = 0.709, 95% CI: 0.603-0.803; p < 0.001). The optimal cutoff value was 3.0 mm; at this value, sensitivity was calculated as 75.7% and specificity as 53.2% (Table 6, Figure 3). 

**Table 6:** Receiver operating characteristic (ROC) analysis AUC: area under the curve

Variable	AUC (95% CI)	Cut-off (mm)	Sensitivity (%)	Specificity (%)	p	Interpretation
Distal alveolar bone loss for predicting ≥4 mm periodontal probing depth	0.709 (0.603-0.803)	3.0	75.7	53.2	<0.001	Acceptable discrimination

## Discussion

In this study, the effect of the positional characteristics of bilateral mandibular impacted third molars on the periodontal status of the adjacent second molar was investigated by evaluating clinical periodontal measurements and distal alveolar bone level obtained from panoramic radiographs together. The main findings of the study showed that Winter angulation, Pell and Gregory classification, and impaction status did not have a significant effect on maximum periodontal pocket depth and distal alveolar bone loss alone; however, there was a moderate positive correlation between periodontal pocket depth and distal alveolar bone loss, and distal alveolar bone loss was an independent predictor of maximum periodontal pocket depth [17-19].

It has long been known in the literature that impacted third molars can cause periodontal destruction in the adjacent second molar. In particular, it has been reported that partially erupted third molars can increase plaque retention, leading to periodontal pocket formation and alveolar bone loss in the distal region [20,21]. However, there is no consensus among studies regarding which type of impaction poses a higher periodontal risk. In our study, no statistically significant relationship was found between Winter angulation and periodontal parameters. Similarly, in multivariate linear regression analysis, Winter angulation was not found to be an independent predictor of maximum periodontal pocket depth. This finding suggests that third molar angulation alone may not be a determining factor for periodontal destruction. Although there are studies in the literature reporting that mesioangular and horizontally impacted third molars may pose a higher risk for periodontal destruction, periodontal destruction is not solely dependent on angulation; It is reported that the periodontal effects of third molars are shaped by the combined effect of many variables such as age, oral hygiene, eruption status, depth of impaction, and individual host factors [20,21]. Therefore, in evaluating the periodontal effects of third molars, not only Winter's angulation but also other anatomical and clinical features should be considered. The fact that no significant relationship was found in our study can be explained by the sample characteristics and the effect of the aforementioned multifactorial structure.

No significant relationship was found between Pell and Gregory classification and periodontal pocket depth or distal alveolar bone loss in univariate analyses. However, in multivariate linear regression analysis, Pell-Gregory Class III impaction was found to be independently associated with maximum periodontal pocket depth. While this may seem like an unexpected result at first glance, it could be related to the fact that Class III teeth are located deeper in bone and have less contact with the oral cavity, thus being less exposed to plaque retention and periodontal inflammation [22,23]. In contrast, Class I teeth, and especially partially erupted teeth, have more contact with the oral environment, which may facilitate periodontal inflammation. This finding suggests that periodontal destruction cannot be explained solely by third molar angulation; anatomical features such as impaction depth, relationship with the ramus, and degree of contact with the oral cavity should be evaluated together. Therefore, treatment decisions should not rely solely on third molar position but should also consider individual periodontal status and other patient-related risk factors. However, the fact that the multivariate regression model created explains approximately 21% of the variance in periodontal pocket depth (R² = 0.212) suggests that periodontal status is a multifactorial process influenced not only by the anatomical variables evaluated in this study, but also by oral hygiene, microbial load, host response, and other individual factors [24].

One of the most important findings of our study is the presence of a moderate positive correlation between maximum periodontal pocket depth and distal alveolar bone loss. The fact that clinical findings obtained through periodontal probing correlate with the alveolar bone level observed on panoramic radiographs demonstrates that clinical and radiographic evaluations complement one another [19]. These findings further support the clinical relevance of radiographic bone level assessment in periodontal risk evaluation.

The fact that ROC analysis showed acceptable discriminative performance in predicting a periodontal pocket depth of ≥4 mm for distal alveolar bone loss (AUC = 0.709) suggests that panoramic radiographs can be helpful not only for anatomical assessment but also in determining periodontal risk. However, it should also be considered that panoramic radiographs, being a two-dimensional imaging method, may not fully reflect the actual bone loss [25].

One of the strengths of this study is that all clinical periodontal measurements were performed by the same researcher using the same periodontal probe, and all panoramic radiographs were obtained with standard imaging parameters. Furthermore, the inclusion of only systemically healthy individuals without a diagnosis of periodontitis reduced the impact of confounding factors such as age and periodontal disease [26]. In addition, thanks to the simultaneous evaluation of the right and left mandibular third molar regions in the same individual, the study provided a comprehensive examination of the periodontal effects of third molar position with a larger dataset obtained from bilateral observations.

However, the study has some limitations. Firstly, since the study has a cross-sectional design, establishing a causal relationship is not possible. Assessments were performed using only panoramic radiographs; three-dimensional imaging methods were not used. Cone-beam computed tomography (CBCT) may provide a more accurate assessment of the relationship between impacted third molars and adjacent periodontal structures and should be considered in future studies. Because linear measurements in panoramic radiographs can be affected by image magnification, distortion, and anatomical superposition, millimeter-level measurement differences may have occurred. In addition, environmental and behavioral factors that may affect periodontal status, such as individual oral hygiene habits, plaque index, brushing frequency, socioeconomic status, dietary habits, and occlusal trauma, were not evaluated. Future studies should also include clinical oral hygiene parameters, such as plaque index and gingival index, to better evaluate their potential influence on periodontal outcomes. Since these variables can affect periodontal health, they may have created residual confounding. Furthermore, since periodontal assessments were performed at a single point in time, periodontal changes and disease progression over time could not be evaluated. Also, because bilateral measurements were obtained from each participant, observations may not have been completely independent. Advanced clustered-data methods, such as generalized estimating equations or mixed-effects models, were not applied because of the limited sample size. Therefore, the findings should be interpreted with appropriate caution, and future prospective studies should use statistical approaches that account for within-individual correlation. Finally, the fact that the study was conducted in a single center may limit the generalizability of the results [27,28]. In addition, intraobserver reliability was not formally assessed, which may have introduced a small degree of measurement variability despite the use of a standardized examination protocol.

Our findings indicate that the periodontal effects of impacted third molars cannot be explained by Winter angulation alone; factors such as the depth of impaction and anatomical position must be evaluated together. Conducting clinical periodontal evaluations in conjunction with radiographic examinations may contribute to more accurate clinical decision-making in the treatment planning of impacted third molars.

## Conclusions

This study demonstrated that the positional characteristics of impacted mandibular third molars alone did not significantly affect the periodontal status of the adjacent second molar. However, a significant positive correlation was found between distal alveolar bone loss and maximum periodontal pocket depth, and distal alveolar bone loss was identified as an independent predictor of periodontal pocket depth. Our findings suggest that the periodontal effects of impacted third molars should not be evaluated solely based on tooth position; combining clinical periodontal examination with radiographic evaluation contributes to more accurate treatment planning. Further multicenter prospective studies with larger sample sizes are needed to confirm these findings.
